# Enhanced Mechanical Properties of 3D-Printed Glass Fibre-Reinforced Polyethylene Composites

**DOI:** 10.3390/polym17091154

**Published:** 2025-04-24

**Authors:** Jan Sezemský, Gregor Primc, Taťana Vacková, Zdeňka Jeníková, Miran Mozetič, Petr Špatenka

**Affiliations:** 1 Department of Materials Engineering, Faculty of Mechanical Engineering, Czech Technical University in Prague, 160 00 Prague, Czech Republic; 2 Department of Surface Engineering, Jozef Stefan Institute, 1000 Ljubljana, Slovenia; gregor.primc@ijs.si (G.P.); miran.mozetic@plasmadis.com (M.M.)

**Keywords:** 3D print, polyethylene, plasma modification, adhesion

## Abstract

Optimisation of the tensile strength of thermoplastic polymer-matrix composites remains a scientific as well as technological challenge for 3D printing technology due to the mass application of composite materials. Inadequate mechanical properties are due to the mismatch in the surface energies of the polymer and fillers. In this study, an additively manufactured composite was 3D-printed and tested. The composite consisted of a linear low-density polyethylene matrix filled with glass fibres. Composite filaments were extruded from neat and plasma-treated polymer powders. Plasma was sustained in oxygen at 100 Pa by a pulsed microwave discharge, and 250 g of polymer powder of average diameter 150 µm was placed into a dish and stirred during the plasma treatment. The O-atom density at the position of the dish containing polymer powder was about 2 × 10^21^ m^−3^, and the treatment time was varied up to 30 min. A gradual improvement in the composites’ tensile and flexural strength was observed at the plasma treatment time up to about 10 min, and the mechanical properties remained unchanged with prolonged treatment time. The tensile strength of composites prepared from plasma-treated polymer increased by one-third compared to those based on untreated powder. However, reinforcing the modified polyethylene with plasma-treated glass fibres did not result in further significant mechanical improvement compared to untreated fibres. In contrast, strength values doubled using glass fibres with silane sizing in combination with plasma-modified matrix. The results were explained by the increased surface energy of the polymer powder due to functionalisation with polar functional groups during plasma treatment.

## 1. Introduction

3D printing of polyolefins remains a technological and scientific challenge [1]. Because of polyolefins’ non-polar character and high crystallinity, researchers have reported issues like high warpage, shrinkage, insufficient adhesion to the printing bed, and poor interlayer adhesion [2,3]. For some applications, the use of polyolefins can be an obstacle, considering their inadequate mechanical properties. Due to the absence of reactive groups in their polymer chains, the interfacial adhesion with many materials is hard to achieve, let alone control [4].

The incorporation of various fillers to improve the properties and performance of printed polyethylene (PE) and polypropylene (PP) composites has become an attractive scientific topic in recent years. Besides classical materials, i.e., glass in the form of short fibres [5,6], powder [7], and spheres [8], other fillers like carbon fibres [9], carbon nanotubes [10], and inorganic nanofillers [11,12] have been used. Organic fillers such as cellulose [13,14], natural fibres [15,16,17,18], and waste material [19,20] are also becoming more popular for the development of new sustainable materials. In the majority of cases, only the elastic modulus increased after reinforcement, however, other mechanical properties like strength and ductility dropped significantly or did not reach the values of the base material. The reasons might be the presence of defects in the printed composite and poor interfacial adhesion between the matrix and the reinforcement. Casamento et al. [10] used PP grafted with maleic anhydride to improve the adhesion between PP and carbon fibres (CF) or carbon nanotubes (CNT). A better interaction between the fillers and the matrix was observed. The tensile strength increased by 29% and the modulus increased by four times when CFs were incorporated into the grafted PP. On the other hand, no relevant improvement in the tensile strength was achieved in such a composite, probably because of CNT agglomeration. Stoof et al. [18] used malleated polypropylene as a coupling agent and treated several types of natural fibres in an alkali solution. The tensile strength of the neat-printed polypropylene was about 17 MPa. It increased up to 22.45 and 23.55 MPa for composites with 30% of hemp or harakeke fibres, respectively. Pre-consumer PP/gypsum powder composites were also tested, but only a small increase of 9% in tensile strength relative to neat PP was observed. Tarrés et al. [17] prepared a biocomposite consisting of biobased polyethylene and thermomechanical pulp fibres. Maleic anhydride functionalised PE was used as a compatibiliser. Many cracks and holes were reported in the samples printed from the neat material. However, the addition of fibres improved internal adhesion between the layers of the subsequently printed materials. Therefore, a significant improvement in tensile strength and modulus values of the printed composite was obtained by the incorporation of pulp fibres.

The brief state-of-the-art indicates better interphase adhesion between the printed polymers and the incorporated fillers using chemical compatibilisers. However, the chemical methods for the treatment of either polymers or fillers are often regarded as ecologically hazardous and harmful to health, thus, an alternative would be useful in modern industry.

Plasma treatment is an established technology used for improving the adhesion properties of polymers [21]. The foundations of plasma technologies for the treatment of organic materials are presented in [22]. Briefly, non-equilibrium gaseous plasma is a source of positively charged ions, neutral radicals with significant potential energy [23], and energetic photons capable of breaking bonds in the surface film of polymer materials [24]. Plasma treatment will make any polymer wettable, including polytetrafluoroethylene [25]. The surface finish of plasma-treated polymers depends on the fluences of all reactive species, and the optimal fluences depend on the type of polymer [26]. It is well-known that during plasma modification, new functional groups appear on the treated surface and thus affect its surface energy and wettability [27,28]. The surface functionalisation with polar groups is not permanent, but hydrophobic recovery is usually observed for all polymers, including polyethylene [29].

Numerous studies have explored the influence of plasma treatment on the interfacial properties of polymer composites but have predominantly focused on fibre surface modification. Plasma modification introduces polar functional groups (e.g., –OH, –C=O, –COOH, –NH_2_) and increases surface roughness, both of which enhance fibre wettability and mechanical interlocking with the matrix. For example, Liu et al. [30] demonstrated significant increases in fibre surface roughness and corresponding improvements in interlaminar shear strength after plasma treatment of carbon fibres. Similar enhancements have been reported across both thermoset and thermoplastic-based composites [31,32,33,34,35,36,37,38]. Interestingly, even non-polar matrices like polypropylene and polyethylene benefited from plasma-treated fibres, showing tensile strength improvements of up to 20–48% [39,40]. However, certain studies also reported negative outcomes, where plasma exposure led to damage of the fibre’s surface and thus reduced mechanical performance [41,42].

An alternative, yet less explored, approach involves using plasma modification of the polymer matrix. Couto et al. [43] treated polypropylene powder and agave fibres with oxygen plasma and assessed the mechanical properties of the resulting composite. Despite the successful incorporation of oxygen-containing functional groups such as –OH, O–C, C=O, and –COOH on the polypropylene surface, the injection molded composite exhibited mechanical properties nearly identical to those of untreated ones. This was attributed to the melting phase and extensive mixing of the polymer during processing, which likely led to the loss of surface-bound functional groups. Similarly, Anjumol et al. [44] reported only a modest 6% increase in tensile strength for composites made from plasma-treated polyethylene and 10% banana fibres, with even less improvement observed at higher fibre concentrations. The use of melt blending likely caused a reduction in polar groups due to shear and thermal effects in the molten polymer. In contrast, Sari et al. [45] and Keerthiveettil et al. [46] demonstrated that plasma treatment of polyethylene powder led to significantly enhanced interfacial adhesion and mechanical performance, up to 20% improvement in tensile and flexural strength of polymer composites processed by rotational molding. The low-shear, pressure-less nature of rotomolding minimised the loss of functional groups during processing, allowing them to contribute more effectively to interphase adhesion. The direct dry mixing of plasma-modified polymer powder with fibres, without creating intermediate melt-blended materials, preserved surface functionalities and further improved the composite’s strength.

In this article, we report on a method for synthesising polyolefin composites used in 3D printing. We treated the polymer powder with non-equilibrium oxygen plasma before synthesising the composite and observed a significant increase in the tensile and flexural strengths of printed polyethylene reinforced with glass fibres.

## 2. Materials and Methods

### 2.1. Materials

In this work, the powdered linear low-density polyethylene (PE) Dowlex™ 2629.10UE (Dow Chemical Company, Midland, MI, USA) was used as a matrix for the synthesised composites. The powder was of irregular shape, and the average lateral dimension was about 150 µm. Short glass fibres (GF) MF 7980 (Lanxess AG, Cologne, Germany) of medium diameter of 14 μm and length of 190 μm served as fillers. This is a DIN1259 aluminium borosilicate glass with an alkali content of less than 2% by weight. The same type of fibres, labelled MF 7982, were coated by the manufacturer (sGF), and were used for comparison. These materials were used to synthesise glass-fibre-reinforced polyethylene (GFRPE) composites.

### 2.2. Synthesis of Composite Materials

**Figure 1:**
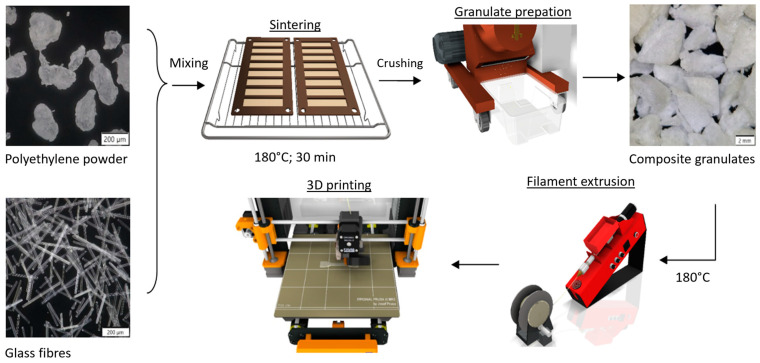
Material flow of prepared composite specimens.

The testing specimens were prepared by fused deposition modelling (FDM) 3D printing. The technological process of filament fabrication consisted of several steps, which are schematically described in Figure 1. At first, the polyethylene powder and glass fibres were dry mixed in the desired weight ratio to obtain a uniform distribution of fillers in the matrix. For one coil of filament, 250 g of raw material was used in total. This mixture was sintered in the oven at atmospheric pressure and 180 °C for 30 min. Thereafter, it was cooled to ambient temperature in a laboratory environment (room temperature, 30–50% relative humidity). The sintered plates were then crushed into pellets using a laboratory shredder machine. The composite filaments were produced from the pellets using the Noztek Pro (Noztek, Shoreham-by-Sea, UK) single-screw extruder. The extrusion speed was 2.5 m per minute, and the temperature was set at 180 °C. Due to the abrasive effects of the glass fibres, a steel nozzle with a 1.75 mm diameter was used. Finally, the composite samples were printed on the 3D-printer Prusa i3 MK3S+ (Prusa Research a.s., Holešovice, Czech Republic), and a spring steel sheet with smooth double-sided polyetherimide (PEI) was used as the build plate to avoid printed specimens detaching. The GCode files were generated by the PrusaSlicer 2.3.3 software.

### 2.3. Testing Methods

The 3D-printed samples were characterised for their mechanical properties and microstructure and were analysed for their thermal and chemical properties. Tensile strength and ductility were measured on prepared composite filaments and tensile test specimens with dimensions according to ASTM D638 (Type IV) [47]. A speed of 50 mm·min^−1^ was used in the tensile testing process. Flexural strength was determined by the three-point bending test using ASTM D790 [48]. The distance between the supports was set to 60 mm, and the test speed was 10 mm·min^−1^. Both tensile and flexural tests were performed on a universal testing machine, Exceed E42 (MTS Systems Corporation, Eden Prairie, MN, USA). The mechanical properties of the printed GFRPE composite were obtained by testing 10 specimens in each series.

The chemical composition of untreated and plasma-treated polyethylene powder was characterised by X-ray photoelectron spectroscopy (XPS). All powder samples were pressed into an Indium foil, and the signal was removed from the spectra. The XPS spectra of the powder surfaces were measured in a TFA XPS spectrometer (Physical Electronics, Chanhassen, MN, USA) with a hemispherical analyser. Monochromatic Al Kα radiation at the photon energy of 1486 eV was used to excite the electrons of the PE surface. The take-off angle was 45° from the sample surface. Survey-scan spectra were obtained at a pass energy of 187 eV using an energy step of 0.4 eV. The high-resolution C1s spectra were acquired at a pass energy of 23.5 eV and a 0.1 eV energy step. The quantification of elements was performed by the built-in MultiPak software 9.9.0.

The effect of plasma treatment on the thermal behaviour of modified PE was examined using differential scanning calorimetry (DSC) with the STA 409 PG Luxx device (Netzsch GmbH & Co. Holding KG, Selb, Germany). Three temperature cycles corresponding to the sample preparation process were measured. A scanning electron microscope (SEM), JEOL JSM-7600F (Jeol Ltd., Tokyo, Japan), was used to observe the surface morphology of fractured composite samples. The specimens were broken by cryogenic fracturing in liquid nitrogen. The fractured surfaces were sputtered with a Cu layer by vacuum sputter coater HVM Flexicoat 3 (HVM PLASMA spol. s r.o., Prague, Czech Republic) and analysed by SEM with an accelerating voltage of electrons 5 kV using the secondary electron detector.

### 2.4. Plasma Treatment

**Figure 2:**
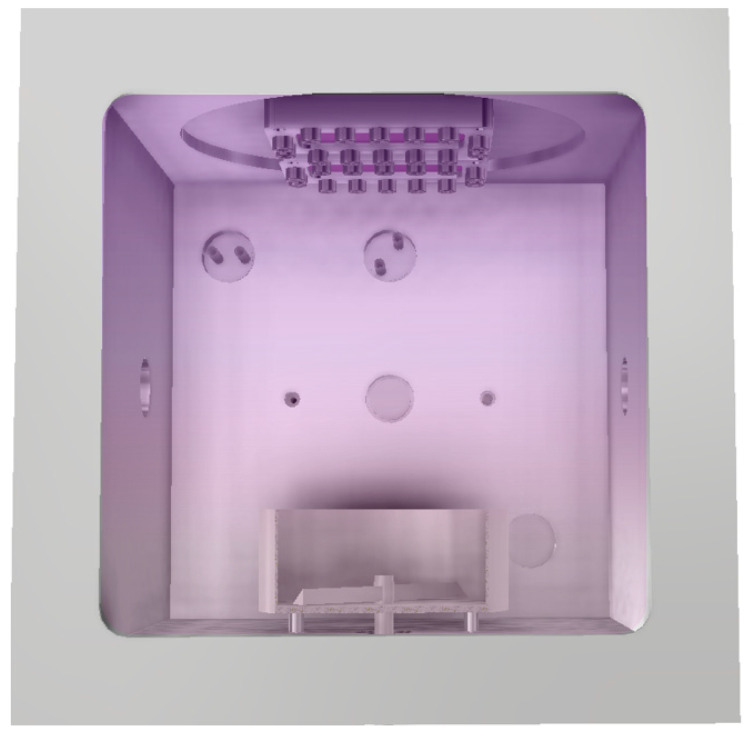
The inside view of the LA 400 reactor chamber for plasma modification.

The modification of the polyethylene powder was performed using a low-pressure plasma laboratory device LA 400 (SurfaceTreat, a.s., Turnov, Czech Republic). The properties of this reactor are described in detail elsewhere [49]. The reactor is schematically shown in Figure 2. It is of cubic shape with a lateral dimension of 40 cm. A microwave generator operating at the standard industrial frequency of 2.45 GHz is mounted on the upper flange, and a dish with polymer powder is on the bottom of the plasma reactor. The reactor is pumped with a two-stage rotary vane pump Duo 65 (Pfeiffer Vacuum, GmbH, Aßlar, Germany). The reactor is first pumped down to the ultimate pressure, which is about 1 Pa. Oxygen of commercial purity of 99.9 vol.% is introduced through a flow controller during continuous pumping. The oxygen flow of 300 sccm ensured a constant pressure of about 100 Pa in the plasma reactor. Gaseous plasma is an electrically conductive medium, so the electromagnetic field of the microwave generator did not penetrate deep into the plasma due to the skin effect. That is why the dense plasma was only next to the upper flange, and the remaining volume was occupied by the diffusing plasma of low luminosity. Molecular oxygen is partially ionised and dissociated in the dense plasma. The reactive oxygen species diffuse over the reactor and eventually reach the surfaces. The reactor is made from aluminium. The charged particles neutralise on the aluminium (or any other) surface at a very high probability, while the probability for heterogeneous surface recombination of oxygen atoms to O_2_ molecules is only about 0.0001 on an oxidised aluminium surface at room temperature [50]. The differences in the loss rate cause a large ratio between the dissociation and ionisation fractions at the bottom of the reactor, where the dish with polymer powder is placed. The major reactants are thus neutral oxygen atoms, which are capable of forming polar functional groups on the surface of polymer powder without affecting other polymer properties. To achieve uniform treatment of the PE powder, the dish is equipped with a mixing device, which rotates at a constant frequency of 40 rpm within the apparatus. In one plasma modification cycle, a total of 250 g of raw material (polymer powder) was weighed and modified. The plasma treatment time was up to 30 min.

### 2.5. Printing of the Synthesised Composites

Due to contradictory information about printed PE-based composites, the first step was an optimisation of printing parameters, namely, nozzle temperature and printing speed. The nozzle temperature was varied between 180 and 260 °C, and the printing speed was between 20 and 100 mm∙s^−1^. The longitudinal-oriented infill specimens, which were defined by a 0° angle between the laid filament and the x-axis of the 3D printer, were prepared. The whole experimental procedure with set parameters is summarised in Table 1.

## 3. Results and Discussion

### 3.1. XPS Analysis

**Figure 3:**
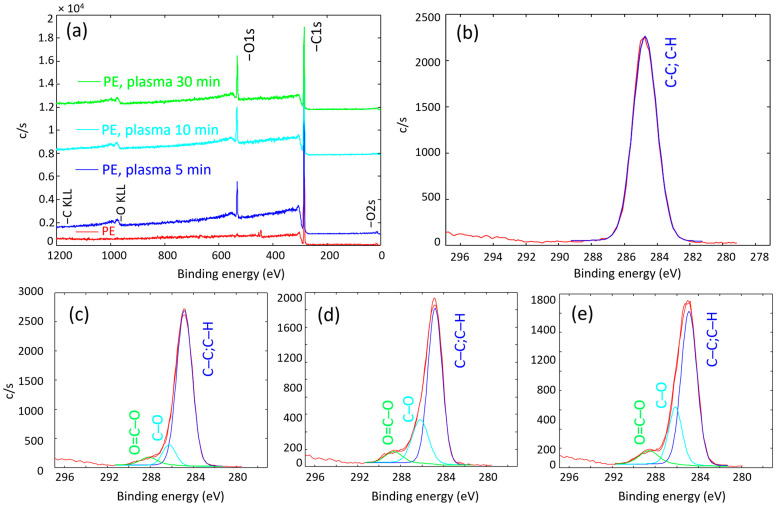
XPS survey spectra for an untreated PE and modified powders (**a**) and high-resolution C1s spectra for an untreated PE (**b**); 5 min treated (**c**); 10 min treated (**d**); and 30 min treated PE powder (**e**).

The surface of the PE powder was analysed using the XPS method. The elemental and chemical compositions were compared for untreated PE and plasma-modified PE (marked as tPE) with different treatment times in oxygen plasma, i.e., 5, 10, and 30 min. The plasma-treated samples contained carbon and oxygen in the surface layer, as illustrated in Figure 3a. The atomic concentrations of individual elements were calculated from the integrated intensities of C1s and O1s photoelectron spectra measured in high-resolution mode. The XPS survey spectra indicated 3 at.% of oxygen on the surface of untreated PE powder and 11, 16, and 20 at.% of oxygen for treatment times of 5, 10, and 30 min, respectively. Carbon was presented in three different chemical states, in bonds C-C or C-H, C-O, and O=C-O, as shown in Figure 3b–e and summarised in Table 2. It should be noted that the high-resolution C1s peak of an untreated sample did not reveal a measurable large concentration of any bonds but C-C or C-H, so oxygen was not chemically bonded to carbon and was probably present on the sample as an impurity. In any case, the concentration of oxygen-containing functional groups significantly increased after the plasma modification of polyethylene powder. Newly bound groups can improve surface energy and thus influence its polarity. This effect is beneficial for adhesion improvement between phases of composites [46].

### 3.2. DSC Analysis

**Figure 4:**
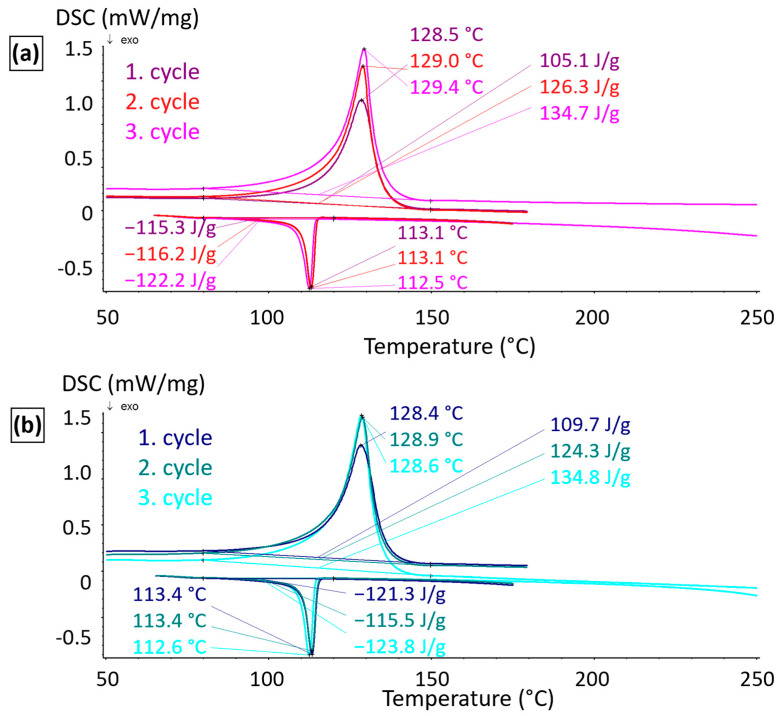
DSC curves of untreated PE (**a**) and plasma-treated tPE (**b**).

The DSC analyses of the neat and plasma-treated PE were performed to test any modification of the bulk polymer properties after plasma treatments. The polymer powder was treated with oxygen plasma in the reactor shown in Figure 2 for 10 min. The DSC thermographs of the untreated and plasma-modified PE matrix are presented in Figure 4. Three heating cycles were measured. In the first and second cycles, the material was heated up to 180 °C, which corresponded with the sintering of the composite mixture and filament extrusion. In the third cycle, the material was heated to 260 °C. This was the maximum temperature that could be reached in the nozzle of our 3D printer. The graphs in Figure 4a for untreated and Figure 4b for plasma-treated PE show almost identical behaviour of both materials. The melting temperature (Tm) was approximately 129 °C, and the crystallisation temperature (Tc) was around 113 °C for both the neat and modified polymer samples in all three cycles. No changes in the structure, such as degradation or crosslinking, which would affect the character of the measured curves, could be observed. As mentioned above, the changes might have occurred due to bond breakage caused by extensive irradiation with energetic photons from gaseous plasma [51]. Plasma treatment affected only surface chemical composition and therefore, composites could be prepared from both the untreated and treated PE using the same technological process without any undesirable influence on thermal history or mechanical properties.

### 3.3. Filament Properties

**Figure 5:**
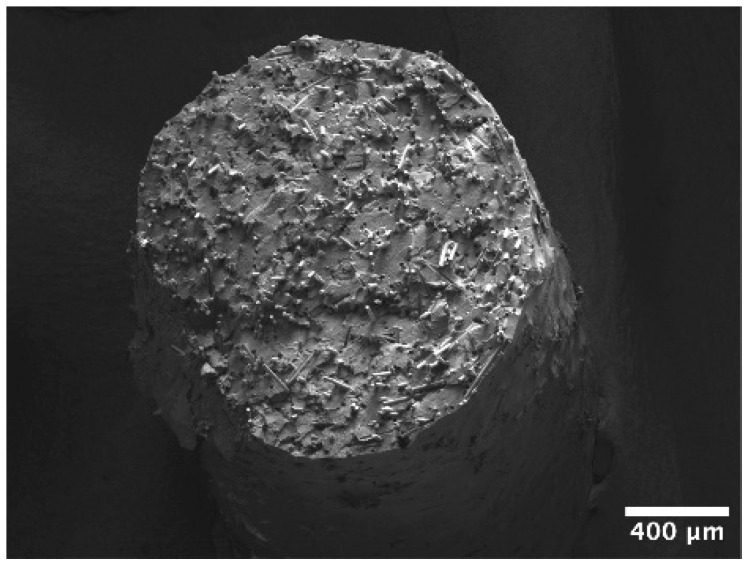
SEM micrograph of tPE-GF30 filament.

The structure and properties of extruded filaments from (un)treated polyethylene and glass fibres were evaluated in the first stage. Figure 5 depicts the fracture surface of the filament consisting of 30 wt.% GF and tPE matrix (tPE-GF30). The presence of voids and pores, which would potentially negatively affect the mechanical properties of composite prints, was not found. The fibres used for reinforcement were uniformly distributed in the matrix, without signs of agglomeration. The fibres were also apparent on the external surface, where they may cause abrasive degradation of printing nozzles.

**Figure 6:**
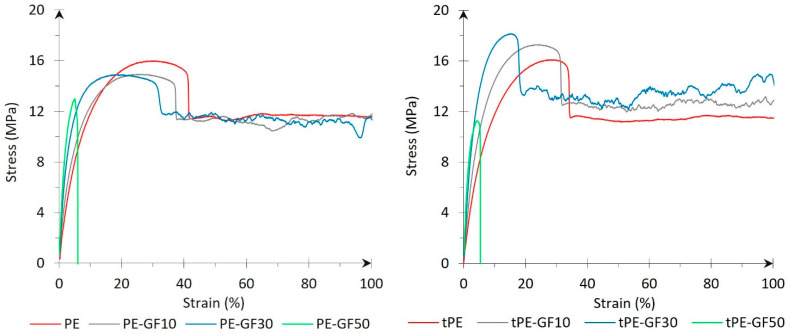
Stress-strain curves of filaments containing unmodified PE and plasma-treated tPE with different concentrations of GF.

A tensile test was performed on the filaments containing 10, 30, and 50 wt.% of glass fibres and filaments free from fibres for both (un)modified states of PE. Stress-strain curves in Figure 6 provide insight into material behaviour for different filament compositions. The presence of rigid fillers in the matrix limited the movement of polymer chains, resulting in a steeper beginning of the loading regime. Higher stiffness eliminates problems with filament buckling, which may occur during printing with flexible polymers such as neat polyolefins [52]. The necking, followed by intensive plastic deformation, appeared on the unfilled filaments and those reinforced by 10 and 30 wt.% GF. The material did not break even after reaching a deformation of 100%. A sharp decrease in ductility and ultimate strength was observed for the 50 wt.% GF concentration. The matrix was oversaturated by fibres, and the composite became brittle since deformation at break reached 5%. A significantly different trend of ultimate tensile strength was found if glass fibres were incorporated into a neat or plasma-treated PE matrix. Composite filaments synthesised from untreated PE showed tensile strength reduction in comparison to unfilled PE. On the contrary, tensile strength increased by filling plasma-treated tPE up to 30 wt.% GF. This result indicated that plasma modification can improve interfacial adhesion between used phases, which was further verified on the FDM printed samples in the next sections.

### 3.4. Optimisation of Printing Parameters

The influence of process parameters on the mechanical properties was evaluated in trial prints. For this optimisation, a filament consisting of a matrix made from plasma-treated tPE powder and 30 wt % of glass fibres was used, and composite testing specimens with 0°orientation were printed and analysed. In this set of experiments, the nozzle temperature and the printing speed were variable.

**Figure 7:**
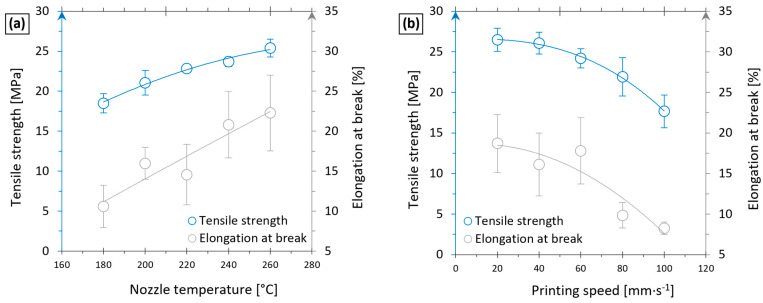
The tensile strength and the elongation at break of tPE-GF30 samples versus the nozzle temperature at a constant printing speed of 60 mm∙s^−1^ (**a**), and versus the printing speed at the constant nozzle temperature of 240 °C (**b**).

**Figure 8:**
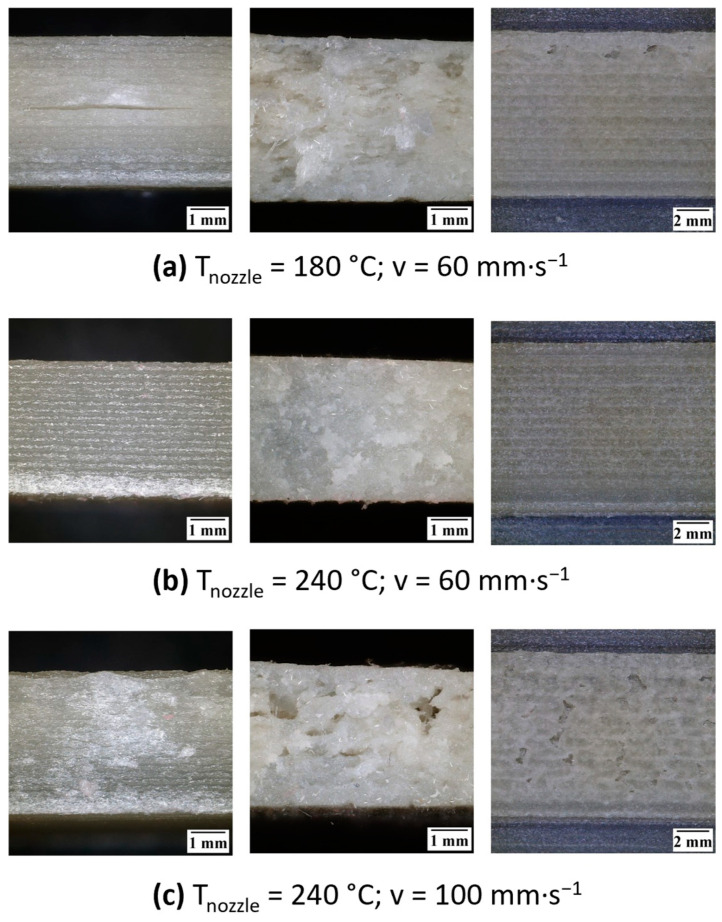
Surface quality of 3D-printed tPE-GF30 samples using insufficient nozzle temperature (**a**); optimal printing parameters (**b**); high printing speed (**c**).

Figure 7a shows a gradual increase in the tensile strength of printed composite from 18.5 to 25.4 MPa in the temperature range of 180 to 260 °C. Higher printing temperatures also resulted in a larger ductility, where the elongation at break increased from about 10 to 17%. The explanation of these improvements is revealed through the optical microscope; images are depicted in Figure 8. By setting a too-low printing temperature, the formation of gaps between printed beads was observed due to insufficient joining of particular layers (Figure 8a), resulting in a reduction of the mechanical performance. At higher printing temperatures, polymer chains may easily diffuse across the boundary of individual layers, leading to adequately connected rasters with no print-induced voids [53]. Thus, good quality of both the inner and outer surface of printed samples (as shown in Figure 8b was achieved by setting the nozzle temperature to 240 °C.

On the other hand, the printing speed had the opposite effect on strength and ductility. A gradual decrease in both the tensile strength and the elongation at break was observed as the printing speed increased, as presented in Figure 7b. At high speeds, a sufficient amount of material is not supplied through the extruder to create the intended layers of the printed object [54]. Tearing and improper formation of laid layers resulted in a poor quality of printed composites, as shown in Figure 8c, leading to a reduction in mechanical properties.

Based on the results summarised in Figure 7, the optimal printing parameters were deduced. Concerning the printing time, the printing speed should be as low as possible. However, the printing speeds in the range between 40 and 60 mm∙s^−1^ provide reasonable tensile strength and ductility concerning the total printing time. This speed interval is significantly higher than the recommended values in publications [7,55], where authors reported extensive problems with filament buckling at printing speeds above 5 mm∙s^−1^. High printing temperatures limit the formation of structural defects, but should be controlled to prevent possible degradation of the PE matrix due to excessive heat in the nozzle. Based on the provided optimisation, composite samples were printed, setting the nozzle temperature to 240 °C and a printing speed of 60 mm∙s^−1^ in further experiments.

### 3.5. Effect of Matrix’s Plasma Treatment

**Figure 9:**
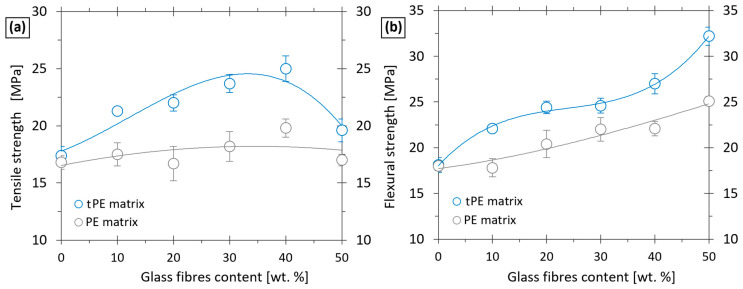
Tensile strength (**a**) and flexural strength (**b**) dependence on the amount of GF and the use of matrix plasma treatment in a composite print.

Figure 9 represents the influence of plasma treatment of PE powder on the tensile (a) and flexural (b) strengths of the printed composites with different concentrations of glass fibres. In the case of unfilled PE printed samples, both tensile and flexural strengths were not affected by plasma treatment, which is consistent with the results of the DSC analysis. But after reinforcement with a specific amount of glass fibres, plasma modification played a significant role in the enhancement of the mechanical properties, as supported by initial tensile tests of prepared filaments. In testing samples that were printed from the plasma-modified tPE, the addition of just 10 wt.% GF resulted in an increase in tensile strength from about 17 to 21 MPa and flexural strength from 18 to 22 MPa, respectively. On the other hand, the contribution of the reinforcement was marginal when using untreated PE as a matrix. In this case, the tensile strength only increased from 16.8 to 17.5 MPa, and the flexural strength even decreased from 18.1 to 17.8 MPa, which is within the limits of the experimental error. A similar trend was also apparent for samples with a higher glass fibre content. For instance, composite samples with 40 wt.% GF exhibited a 26% higher tensile strength when using a plasma-modified PE matrix in comparison with an untreated PE. The difference in flexural strength was 22%.

The results presented in Figure 9 were obtained for printed composites synthesised from plasma-treated polymer powder, wherein the treatment time of oxygen plasma was 10 min. In the next set of experiments, we studied the influence of plasma treatment time on the mechanical properties of printed specimens.

**Figure 10:**
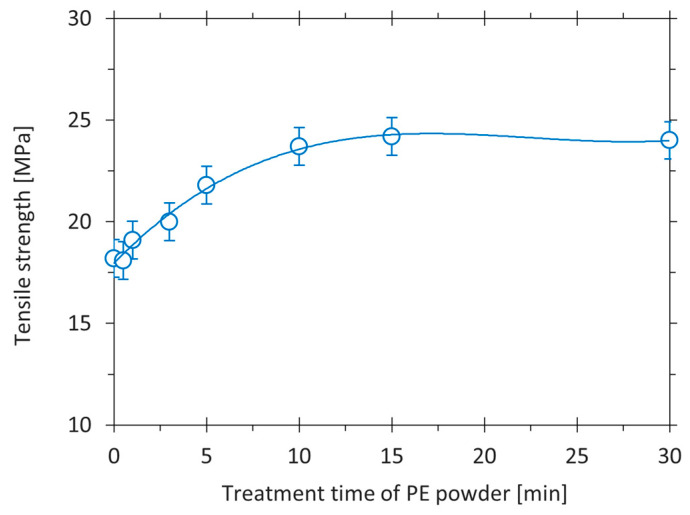
Tensile strength of printed tPE-GF30 composites versus treatment time of used polyethylene powder.

Figure 10 depicts the tensile strength dependence of printed tPE-GF30 composite samples, which were synthesised from PE powder treated for various times in oxygen plasma. A gradual increase in tensile strength appeared in the first few minutes of modification. At around 15 min, the maximal effect of plasma treatment was reached. The tensile strength of printed composites using filaments synthesised from plasma-treated PE powder and 30 wt.% GF achieves the ultimate value of about 24 MPa. Longer plasma-treatment times had no effect on the tensile strength. According to the XPS results provided in Section 3.1, this phenomenon could be attributed to the saturation effect of the PE powder surface with polar oxygen-containing functional groups. On the non-functionalised surface, the number of binding sites is limited, and it increases with plasma treatment time. After prolonged treatment, the maximal concentration of surface polar groups is approached. In our previous study [56], a significant increase in the wettability of PE powder (evaluated by the Washburn method) was observed when a small percentage of oxygen was bonded to the polymer surface. After a 10 min treatment in oxygen plasma, the wettability reached its maximum and did not change with a longer treatment time. Therefore, saturation of the PE surface by polar groups did not affect the ability to interact with reinforcement further, and thus, strength remained unchanged with prolonged treatment time in a printed composite.

**Figure 11:**
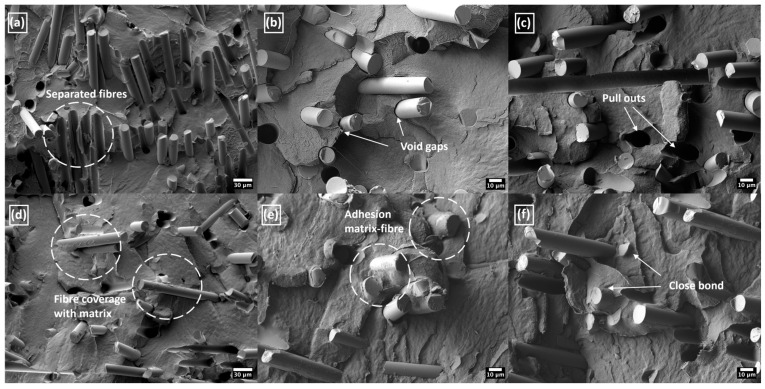
SEM images of printed composites with matrix from untreated PE (**a**–**c**) and plasma-modified tPE (**d**–**f**).

The SEM micrographs shown in Figure 11 confirmed higher interfacial strength for the tPE-based composite in comparison with printed material using an untreated PE matrix. The untreated PE composites revealed many pull-outs and void gaps between the matrix and used fillers, as can be seen in Figure 11a–c. The poor interphase adhesion led to glass fibres breaking out from the surface and their complete separation. This insufficient adhesion was the main reason for the significantly lower strengths observed in the untreated composite samples, as discussed earlier. The micrographs in Figure 11d–f reveal a better interaction for the composition of plasma-treated tPE and glass fibres. In these samples, fibres are locally covered and closely bonded with the matrix, which results in a reduction in the number of void gaps. Also, the improved compatibility between the used phases could limit pull-outs and ensure more efficient stress transfer properties from the matrix to the reinforcement. Overall, increased surface energy and wettability of plasma-treated polymer established a stronger fibre-matrix interface and hence strength values for the printed composites.

### 3.6. Influence of Fillers Modification on Mechanical Properties of Printed Composites

In previous experiments, untreated and plasma-modified polyethylene powders were used as a matrix phase for the synthesis of composite filaments, but the fibres were untreated. Besides that, fibre surface modification is commonly used to enhance the mechanical properties of reinforced polymers. The introduction of polar groups on the fibre surface increases their surface energy, which, in relation to the surface energy of the polymer matrix, improves wettability and consequently enhances adhesion between the matrix and the reinforcement [57]. Therefore, specimens containing unmodified PE and 30 wt.% of treated glass fibres (tGF) in oxygen plasma for 30 min and fibres with a silane surface treatment (sGF) were printed and compared in the final testing setup.

**Figure 12:**
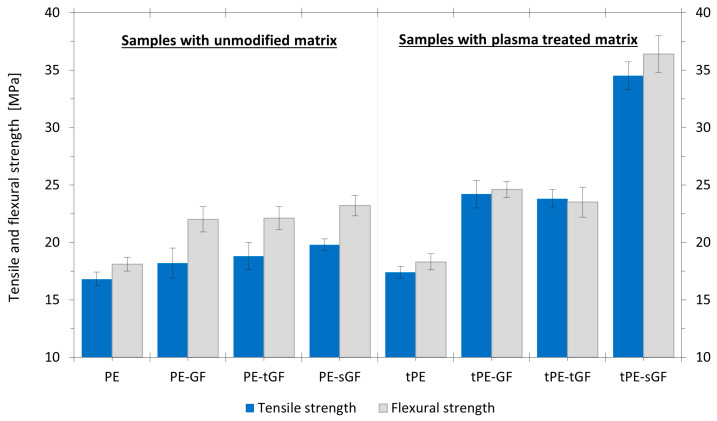
Strength values of composites from untreated (PE) and plasma modified polyethylene (tPE) filled by 30 wt.% glass fibres without any modification (GF), plasma treated (tGF) and silane coated (sGF).

The results of tensile and flexural strengths of 3D printed specimens with different filler compositions are depicted in Figure 12. The strength values of printed composites were basically unchanged by filling neat PE with glass fibres regardless of their modification (untreated or plasma-treated). As discussed earlier, due to the non-polar character of the initially used polymer matrix, it was impossible to achieve sufficient interphase adhesion with filler of opposite polarity. Reinforcing the modified polyethylene with plasma-treated glass fibres also did not result in a significant improvement in mechanical properties. The increase in strength was essentially the same as in the tPE-GF composite containing untreated fibres. Therefore, the primary contribution to the enhancement of mechanical properties in the printed composite came solely from the plasma treatment of the polymer powder used for the matrix. On the other hand, a significant increase in strength values was observed using glass fibres with silane sizing. Filling the plasma-treated matrix with these modified fibres improved both tensile and flexural strength by more than 40%, in contrast to the tPE-GF composite with the untreated fibres and approximately doubled these values compared to the unfilled printed tPE. Such percentual increases are typical for printed composites containing a polar matrix, e.g., polyamide [58,59], not for polyolefins-based prints. The obtained results indicate the potential of using a combination of the matrix’s plasma modification with sizing on the fibres to enhance mechanical properties in printed materials. Our future research will be devoted in more detail to the investigation of various sizing and different types of fibres and their influence on properties in 3D printed composites synthesised from plasma-treated polymers.

**Figure 13:**
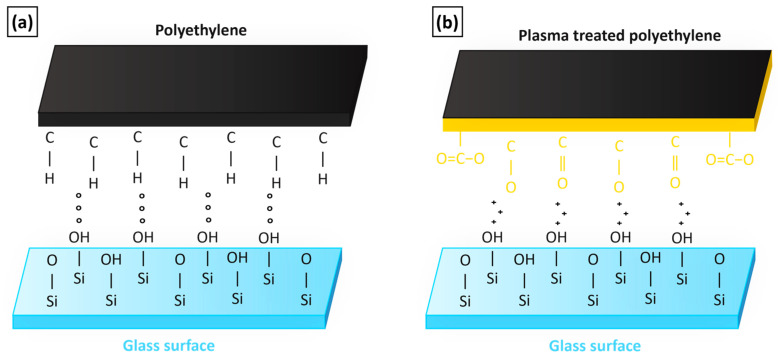
Interaction between unmodified polyethylene (**a**) or plasma-treated polyethylene (**b**) and glass surface in a composite system.

The aforementioned principle of performance enhancement in a printed tPE-GF composite is schematically illustrated in Figure 13. This improvement is attributed to the plasma modification of originally non-polar polyethylene. During plasma treatment, new functional groups (e.g., hydroxyl or carboxyl groups) are introduced onto the treated surface, making it more polar. Due to the amorphous structure of glass, free non-bridging oxygen atoms are present in certain regions, as they do not participate in the formation of the glass network. These oxygen atoms, along with polar groups present in the silane surface layer, have the ability to form stronger intermolecular interactions with the functional groups bonded to the polyethylene, whose surface was plasma-modified [60]. The improved compatibility between the glass fibres and the plasma-treated polyethylene increases the interfacial strength and formation of close bonds, making fibre pull-out more difficult compared to the untreated PE matrix, as previously demonstrated by Weberová et al. [61], where interfacial strength was quantified using the Pin-Collar Strength Test between the glass rod and PE layer. Adhesion to the glass surface increased by 1670% from 0.4 up to 7.0 MPa using plasma-modified polyethylene. Additionally, plasma treatment causes increased surface energy of tPE and thus its higher wettability, as has been experimentally verified in our previous work by Šourková et al. [56], where a substantial wettability enhancement was observed using the Washburn method after powder modification. Further confirmation was provided by Anjumol et al. [44], who measured surface energy based on contact angle analysis, with values increasing from 18.7 to 32.8 mJ/m^2^ and contact angles decreasing from 95° to 78°, for untreated and plasma-treated polyethylene, respectively. By the combination of these positive effects, the modified polyethylene adheres better to the glass fibre surface. In SEM images of fracture surfaces, the remnants of the modified matrix were visible on the fibres in some cases and close bonds between phases reduced voids presence. This effect is beneficial for adhesion improvement between phases of the composite because it enables stress transfer from the matrix to fibres and enhances the bulk mechanical properties of printed composites.

## 4. Conclusions

In this paper, we introduced the ecological alternative to the chemical compatibilisation of printed composites based on a non-polar matrix. The plasma treatment of PE powder was successfully implemented into the composite FDM 3D-printing process. New functional groups were bonded to the originally non-polar surface during plasma modification. Thus, stronger intermolecular interactions are established between the treated matrix and the surface of glass fibres. It was found that the maximal functionalisation and reinforcement effect occurs after 10 min of modification of the PE powder in oxygen plasma. Extending the modification time did not further increase the total amount of bonded polar groups on the polymer surface. Enhanced interphase adhesion proved to be essential for an improvement of mechanical properties. The results showed different performances using (un)treated matrices in printed composites with different concentrations of unmodified glass fibres. An increase of up to 30% in tensile and flexural strengths was achieved in comparison with composites synthesised from untreated polymer powder. The maximal reinforcement effect was observed using glass fibres with silane sizing. Compared to unfilled polymers with a tensile strength of 17.4 MPa and flexural strength of 18.3 MPa, a combination of 30 wt.% of these fibres and modified tPE printed composites resulted in a doubling of strength values to 34 MPa, respectively. 36.4 MPa. Finally, the process parameters for printing with produced composite filaments were optimised. Using the nozzle temperature of 240 °C and printing speed of 60 mm·s^−1^ led to the reduction of internal defects and good surface quality of composite prints.

## Figures and Tables

**Table 1:** Selected parameters of 3D printing.

Variable Parameters		Constant Parameters	
Nozzle temperature	180–260 °C	Bed temperature	90 °C
Printing speed	20–100 mm∙s^−1^	Nozzle diameter	0.8 mm
GF content	0–50 wt.%	Thickness of the 1st layer	0.4 mm
Matrix modification	Neat PE vs. Plasma-treated PE (tPE; O_2_; 10 min; 300 sccm, 100 Pa)	Layer thickness	0.25 mm
Treatment time in plasma	0.5–30 min	Infill orientation	Longitudinal (0°)
		Infill density	100%

**Table 2:** The relative concentrations of the functional groups.

Carbon Bonds [at. %]	C-C, C-H	C-O	O=C-O
PE-untreated	100	0	0
tPE–(5 min)	85.1	9.6	5.3
tPE–(10 min)	69.8	23.9	6.3
tPE–(30 min)	68.0	23.3	8.7

## Data Availability

All supporting data are contained within the manuscript. Further inquiries can be directed to the corresponding author.

## Abbreviations

The following abbreviations are used in this manuscript:

PE	Polyethylene
PP	Polypropylene
CF	Carbon fibres
CNT	Carbon nanotubes
GF	Unmodified glass fibres
tGF	Plasma-treated glass fibres
sGF	Glass fibres with silane sizing
tPE	Plasma-treated polyethylene
GFRPE	Glass-fibre-reinforced polyethylene composite
FDM	Fused deposition modelling
XPS	X-ray photoelectron spectroscopy
DSC	Differential scanning calorimetry
SEM	Scanning electron microscopy
